# Supplementary material for: PhaTYP: predicting the lifestyle for bacteriophages using BERT
Source: Brief Bioinform. 2022 Nov 23;24(1):bbac487. doi: 10.1093/bib/bbac487 (PMC9851330; doi:10.1093/bib/bbac487)
Supplement: Supplementary_file_bbac487 [file supplementary_file_bbac487.pdf]

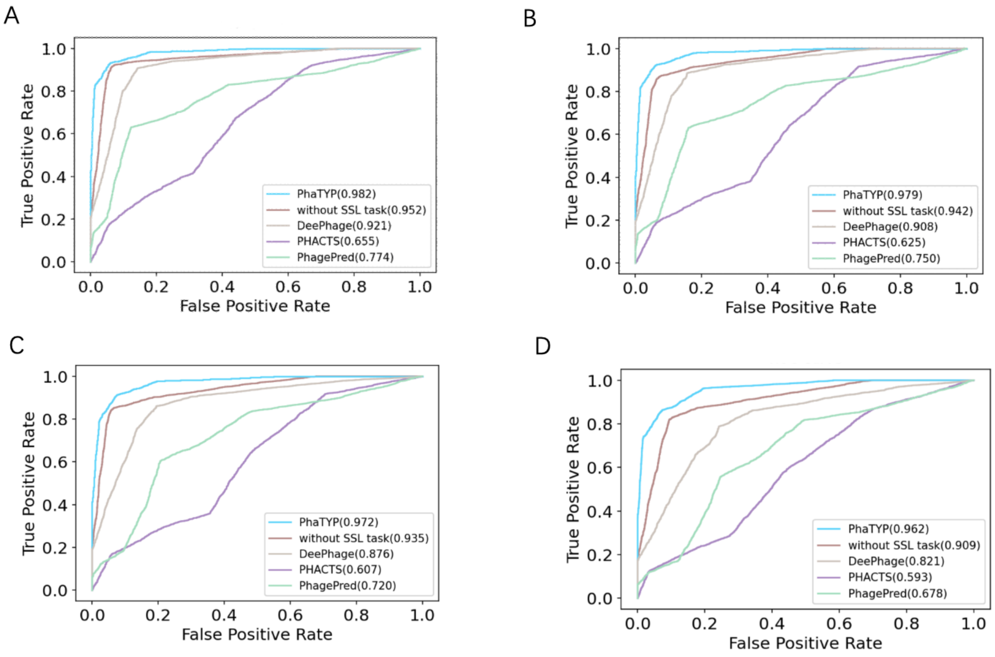

Fig S 1: The ROC curve comparison on the short contigs. The value shown in the legend is AUCROC score. A: 100-400bp contigs. B: 400-800bp contigs. C: 800-1200bp contigs. D: 1200-1800bp contigs.

ROC curves on the short contigs

We generate the ROC curve on short contigs to show the tradeoff between FP rate and sensitivity. The results in Fig S1 reveal that PhaTYP outperforms the other tools across different length ranges. In addition, with the decrease of the length, the gap between PhaTYP and other methods becomes larger, indicating that PhaTYP can perform better on short contigs.

Statistical analysis (p-value) of the classification performance

In order to evaluate whether the performance difference from these results is statistically significant, we calculate the p-value of the ROC curves between two classifiers following the definition in [1]. The results are listed in Table S1.

	DeePhage	PhagePred	PHACTS	BACKCLIP	Without SSL
Complete genome	$<$1e-6	$<$1e-6	$<$1e-6	7.6e-6	0.002
1200-1800	$<$1e-6	$<$1e-6	$<$1e-6	-	$<$1e-6
800-1200	$<$1e-6	$<$1e-6	$<$1e-6	-	$<$1e-6
400-800	$<$1e-6	$<$1e-6	$<$1e-6	-	$<$1e-6
100-400	$<$1e-6	$<$1e-6	$<$1e-6	-	$<$1e-6

Table S 1: Statistical analysis of the classification performance. Because BACKCLIP is not designed to conduct prediction for short contigs, we only record the p-value on the complete genomes.

Table S1 shows the p-values between five methods (including four other tools and ours without SSL) and PhaTYP. As we can see from the table, all the differences are statistically significant, indicating that PhaTYP can achieve better performance in classifying phages' lifestyles.

References

1. James A Hanley and Barbara J McNeil. The meaning and use of the area under a receiver operating characteristic (roc) curve. *Radiology*, 143(1):29–36, 1982.
